# A Tough Pill to Swallow: Demonstration of Esophageal Physiologic Events Explaining Severe Dysphagia Captured on EndoFLIP After LINX Procedure

**DOI:** 10.14309/crj.0000000000001995

**Published:** 2026-02-09

**Authors:** Shay S. Bidani, Alexandria Iakovidis, Lindsey Creech, Manuel Amaris, David Estores

**Affiliations:** 1 Department of Internal Medicine, University of Florida, Gainesville, FL; 2 Department of Gastroenterology, Hepatology, and Nutrition, University of Florida, Gainesville, FL

**Keywords:** dysphagia, EndoFLIP, magnetic sphincter augmentation, LINX device, esophagogastric junction, esophageal distensibility, volume-dependent obstruction

## Abstract

Dysphagia is a common symptom after magnetic sphincter augmentation, a minimally invasive antireflux alternative to laparoscopic Nissen fundoplication. A 69-year-old woman underwent LINX procedure in 2017 for GERD symptoms, after which she had the onset of dysphagia. EndoFLIP demonstrated narrowing and stiffness at lower volumes but acceptable distensibility at the largest balloon volume. The treatment plan aimed to improve swallowing by ingesting larger solid boluses and avoiding additional invasive procedures. This visualization of the obstruction on EndoFLIP presented a more physiologic approach to treatment with ingesting larger, rather than smaller, solid food boluses.

## INTRODUCTION

Dysphagia is a common symptom after magnetic sphincter augmentation (MSA), a minimally invasive antireflux alternative to laparoscopic Nissen fundoplication.^1,2^ MSA involves the implantation of a ring-shaped device of titanium beads known as a LINX device around the lower esophageal sphincter to prevent stomach contents from refluxing into the esophagus.^2^ EndoFLIP is an impedance planimetry test done during endoscopy that measures the dimensions of the esophagus, esophageal contractility, and distensibility index (DI) of the esophagogastric junction (EGJ). EndoFLIP after MSA enables calculation of EGJ-DI, which can help determine whether dysphagia results from a restrictive or fibrotic process related to the LINX device versus the expected distensibility of native tissue.^3^ The expected EGJ-DI cutoff is 2 mm^2^/mm Hg at distention volumes of 50 and 60 mL.^4^ Although prior EndoFLIP studies have reported reduced EGJ distensibility using single-volume measurements, we describe, to our knowledge, the first reported instance of EndoFLIP demonstrating a volume-dependent pattern that correlates with the patient's bolus size–dependent symptoms from LINX-related EGJ restriction.^4,5^

## CASE REPORT

A 69-year-old woman with a history of gastroesophageal reflux disease (GERD), irritable bowel syndrome with diarrhea, and interstitial cystitis presented with severe dysphagia localized to the bottom quarter of her sternum that occurred at least once daily, as well as regurgitation twice weekly. She self-treated her symptoms with aluminum hydroxide and magnesium carbonate with evening meals. She underwent LINX procedure 7 years prior for GERD symptoms and developed new-onset dysphagia within the following year. She underwent esophagogastroduodenoscopy (EGD) with dilation 1 year after LINX placement, as well as cognitive behavioral therapy with speech-language pathology, which improved her symptoms and allowed her to swallow solid foods again. After her initial dilation, she required 2–3 additional dilations, with her most recent 1–3 years before this visit. Per the patient, her symptoms improved for 1 year after each dilation, but she was lost to follow-up until this presentation.

**Figure 1.**
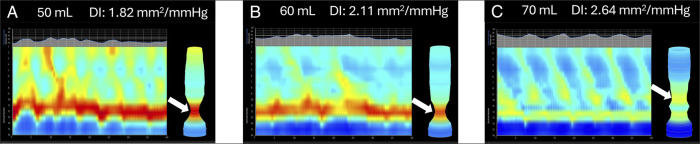
EndoFLIP demonstrating volume-dependent EGJ narrowing and opening after magnetic sphincter augmentation. Arrows indicate the site of EGJ narrowing. (A) Restricted EGJ opening at 50 mL of saline with a low DI (1.82 mm^2^/mm Hg), intrabag pressure of 31.1 mm Hg, and a maximum EGJ diameter of 8.50 mm. (B) Partial improvement in EGJ opening at 60 mL of saline (DI 2.11 mm^2^/mm Hg, 37.9 mm Hg of pressure, and diameter of 10.10 mm). (C) Adequate EGJ opening at 70 mL of saline with preserved repetitive antegrade contractions (DI 2.64 mm^2^/mm Hg, 67.0 mm Hg of pressure, and diameter of 15.00 mm). These findings illustrate a volume-dependent physiologic response that correlated with the patient's bolus size–dependent dysphagia and guided conservative treatment with larger bolus swallows. DI, distensibility index; EGJ, esophagogastric junction.

The patient underwent a timed barium esophagram, revealing a dilated esophagus with residual barium at 1 minute, clearing of barium at the 5-minute mark, and normal passage of a 13-mm barium tablet through the esophagus. Her subsequent EGD showed normal mucosa of the esophagus. High-resolution manometry was not obtained because postoperative changes after MSA can make measurements of lower esophageal sphincter integrated relaxation pressures (LES IRP) and intrabolus pressures (IBP) difficult to interpret. EndoFLIP was instead used to assess EGJ opening and distensibility under graded distention. EndoFLIP demonstrated stiffness at lower volumes, reflected by DI of 1.82 and 2.11 mm^2^/mm Hg at 50 and 60 mL, respectively, but acceptable distensibility at the maximum balloon volume of 70 mL (DI 2.64 mm^2^/mm Hg), indicating adequate opening (Figure 1). Normal contractility was demonstrated by the repetitive antegrade contractions at the highest volume, indicating an acceptable gastroesophageal junction distensibility and opening despite the observed restriction at lower volumes. These findings demonstrate that adequate EGJ opening requires higher distending forces. Therefore, the treatment plan aimed to alleviate symptoms and improve swallowing by ingesting larger solid boluses and allowing a 10-second interval after each swallow. A larger bolus was recommended to increase intraluminal pressure and overcome mechanical restriction from the LINX, thereby mitigating dysphagia symptoms. Avoidance of additional invasive procedures was also recommended. With these recommendations, the patient had improvement in her symptoms.

## DISCUSSION

Up to 67.8% of patients report dysphagia after MSA with a LINX device, with 15.5% reporting long-term symptoms.^3,6^ For patients with persistent dysphagia after MSA, up to 31% undergo endoscopic dilations with an initial success rate of 67%; however, this success rate decreases with repeated procedures.^3^ Roughly 2%–3% of patients with refractory symptoms require device removal with resolution of symptoms ranging from 75% to 100%.^5,7,8^ These invasive postoperative treatments highlight the need for diagnostic tools that can precisely identify the cause of dysphagia and inform targeted treatment strategies that may include avoiding unnecessary operations and complications.

Diagnostic approaches for such cases include the use of EGD, high-resolution manometry, and pH monitoring.^2,3^ Although these tools may provide insight into the pressure dynamics and peristaltic patterns in the esophagus and EGJ, they lack any information on the compliance or distensibility that would help differentiate if the dysphagia is due to mechanical obstruction from the LINX device itself or if it is due to a physiologic abnormality within the patient's EGJ. EndoFLIP offers real-time measurements of the cross-sectional area and pressure in the esophagus thereby allowing calculation of the distensibility index that quantifies EGJ wall compliance. Several studies have explored this in the context of post-MSA dysphagia and demonstrated that a lower DI is associated with dysphagia.^5,9^ Another study used EndoFLIP to optimize the tightness of the device intraoperatively to reduce postoperative dysphagia.^10^ However, these studies focused only on the static measurements by only measuring the DI without correlating the differences in DI at higher FLIP balloon volumes.

Our case addresses this by using various balloon volumes to dynamically correlate EndoFLIP findings with the patient's symptoms, offering a direct physiologic explanation of the patient's symptoms: lower DI at smaller volumes that correlated with the patient's dysphagia with DI normalized at larger volumes. This demonstrates that her dysphagia is volume-dependent and may respond to different strategies for treatment of dysphagia. These findings give clinicians actionable insight, allowing a tailored treatment strategy, such as advising the patient to swallow larger volume solid boluses. In contrast to traditional tools, like EGD, high-resolution manometry, and pH monitoring, which might have prompted repeated dilations or device explanation, EndoFLIP provided a physiologic rationale for a conservative approach, ultimately sparing the patient from further invasive procedures.

Our case highlights the potential for EndoFLIP to refine the diagnostic and treatment approach to post-LINX dysphagia as it offers insights that are not attainable with the more commonly used modalities. EndoFLIP affords an insight into the mechanical and functional causes of dysphagia, which further allows patient-specific treatment strategies and a reduction of unnecessary interventions.

## DISCLOSURES

Author contributions: SS Bidani: drafted the initial manuscript, performed primary literature review, contributed to interpretation of case findings, and prepared revisions. A. Iakovidis: drafted the initial manuscript, performed primary literature review, contributed to interpretation of case findings, and prepared revisions. L. Creech: provided manuscript revisions, refined structure, and ensured clarity of presentation. M. Amaris: contributed to case interpretation, reviewed and edited the manuscript for accuracy, and approved the final draft. D. Estores: supervised case interpretation, provided critical revisions, ensured integrity of clinical content, and approved the final manuscript for submission. David Estores is the article guarantor.

Previous presentation: This case report was presented as a poster presentation at the American Neurogastroenterology and Motility Society (ANMS) August 9, 2025 in Minneapolis, MN.

Informed consent was obtained for this case report.

## ABBREVIATIONS:

DI: distensibility index
EGD: esophagogastroduodenoscopy
EGJ: esophagogastric junction
EndoFLIP: endoluminal functional lumen imaging probe
GE: gastroesophageal
GERD: gastroesophageal reflux disease
IBP: intrabolus pressure
IRP: integrated relxation pressure
LES: lower esophageal sphincter
LINX: magnetic sphincter augmentation device
MSA: magnetic sphincter augmentation
